# The Impact of Land Use Structure Change on Utilization Performance in Henan Province, China

**DOI:** 10.3390/ijerph20054251

**Published:** 2023-02-27

**Authors:** Yanqi Zhao, Yue Zhang, Ying Yang, Fan Li, Rongkun Dai, Jianlin Li, Mingshi Wang, Zhenhua Li

**Affiliations:** 1 Institute of Resources & Environment, Henan Polytechnic University, Jiaozuo 454003, China; 2 Collaborative Innovation Center of Coal Bed Methane and Shale Gas for Central Plains Economic Region, Jiaozuo 454100, China; 3 Collaborative Innovation Center of Coal Work Safety and Clean High Efficiency Utilization, Jiaozuo 454100, China

**Keywords:** land use structure, land use performance, land type conversion, information entropy, grey correlation

## Abstract

Because of the trends in population growth and rapid industrialization and urbanization, the intensity and structure of land use are undergoing great changes. Henan Province is an important economic province and a major grain producing and energy consumption area, and its land use plays a key role in the sustainable development of the whole of China. This study takes Henan Province as the research object, selects panel statistical data from 2010 to 2020, and discusses the land use structure (LUS) in Henan Province in terms of three aspects: information entropy, analysis of land use dynamic change, and land type conversion matrix. Based on the indicator system “social economy (SE)—ecological environment (EE)—agricultural production (AP)—energy consumption (EC)”, a land use performance (LUP) evaluation model was constructed to judge the performance of various land use types in Henan Province. Finally, the relational degree between LUS and LUP was calculated through the grey correlation. The results show that among the eight land use types in the study area since 2010, land used for water and water conservancy facilities increased by 4%. In addition, transport and garden land changed significantly, and was mainly converted from cultivated land (decreased by 6674 km^2^) and other land. From the perspective of LUP, the increase in ecological environment performance is the most obvious, while agriculture performance is lagging behind; it is worth paying attention to the energy consumption performance, which is decreasing year by year. There is an obvious correlation between LUS and LUP. LUS in Henan Province presents a gradually stable state, and the transformation of land types promotes LUP. Proposing an effective and convenient evaluation method to explore the relationship between LUS and LUP is very beneficial in helping stakeholders to actively focus more on optimizing land resource management and decision making for the coordinated and sustainable development among agricultural, socio-economic, eco-environmental, and energy systems.

## 1. Introduction

The land resource is an important spatial carrier of human socioeconomic development [1]. Furthermore, land use and land cover change (LUCC) plays a significant role in regional and global environmental changes [2]. In the past several decades, because of population growth, industrialization and urbanization, climate change, etc., China’s social and economic achievements have been accompanied by major changes in land use structure (LUS), which in turn accelerated some serious consequences, such as spatial sprawl and reduction in vegetation cover, inefficient land use or even degradation, escalating environmental pollution and urban water logging, heat island effects, and an increasingly tense relationship between people and land [1,2]. Therefore, it is of great important to balance land utilization, economic growth, and environmental sustainability in the process of development [3].

The efficiency of LUS is vital for the level of sustainable development [1] and has become a growing concern in urban design, city management, and land use analysis [1,4]. The current studies of LUS focus on LUCC’s spatial-temporal pattern or evolution of land use structure using spatial correlation and statistical models [5,6] or geographic methods [7,8], mechanisms, or driving factors of land use change. For example, using time series Landsat imagery and the Random Forest algorithm, Shen et al. [9] detected the land use conversions of the Huangshui River Basin of China. Using the FLUS model, Xuet al. [10] simulated land use pattern evolution in Jincheng, Shanxi Province. Huang et al. [11] simulated the temporal and spatial characteristics of urban land use in Wuhan from the past to 40 years into the future. Wu et al. [2] and Burra et al. [12] revealed the driving mechanism of land use change. To solve the fragmentation problem in China, Zhang et al. [13] also studied how institutional change affects diverse governance structures of land consolidation. In addition, there are scholars who have studied land use structure optimization [14,15,16], and the relationships between population [17,18] and energy consumption [19] and land use.

The purpose of studying land use structure (LUS) is to improve land use performance (LUP). That is, the premise of the rational layout and optimization of land structure finally ensures that the land use achieves good economic, social, and environmental effects. In terms of industrial land efficiency, Chen et al. [20], Dong et al. [21] and Chen et al. [22] researched the impact mechanism of different ranks of development zones and industrial transformations on land use efficiency. Regarding agricultural land use, Li et al. [23] proposed an agricultural land use decision support system (LDSS) and Ustaoglu et al. [24] developed a spatial model for land suitability assessment to study the comprehensive benefits of land use. For efficiency of urban land use, using Data Envelopment Analysis (DEA) and the Tobit model, Gao et al. [25], Ding et al. [3] and Song et al. [26] estimated the land use efficiency and its possible determining factors for the resource-based cities or urban agglomerations in China. He et al. [27] and Zhang et al. [28] also studied the spatial effects of urban form on land use efficiency and the optimizing model of urban land use structure [4].

Understanding the relationship between performance and different types of land use has been of great significance [17] to optimizing land resource allocation and improving utilization efficiency in order to achieve regional sustainable development [1]. The above researchers have made significant achievements in the related fields of LUCC. Previous research objectives chiefly focused on improving the efficiency of LUS, and optimizing the allocation of land resources and the formulation of land use policies [1], but rarely involved environmental protection and energy consumption. In particular, although some valuable results have been obtained, few studies have paid attention to the correlation between land use structure change (LUSC) and land use performance (LUP), due to the interactions and multiple efficiencies among the ecological environment, social economy, agricultural production, and energy consumption.

Henan Province is an important economic province and a major grain producing and energy consumption area in China, having a large population but less arable land per capita. The degree of land development and utilization has been very high. The comprehensive effect of population, urbanization, and industrialization has created tension around land resources, and significant changes have taken place in land use types and structures over the past decades [7]. In some regions, the contradiction between land supply and demand is acute, environmental problems are prominent, natural disasters occur frequently, and land use efficiency and sustainable ability are reduced. It is difficult to guide the land use system towards a sustainable track if the driving factors and key mechanisms of coordination among the complex coupled systems cannot be scientifically identified [29].

Considering the gap in research and the actual situation of Henan Province, to examine possible policy and measures to attain sustainable land use [1] this study aimed to: (1) clarify the structural change characteristics of eight land use types; (2) evaluate the performance of all types of land use in Henan Province; and (3) precisely uncoverthe relationship between land use structure (LUS) and land use performance (LUP) in Henan Province. So, in this study, an LUP evaluation system was proposed and the relationship between LUS and performance was derived using the grey relational degree for the identification of the core driving factors of land use change. This can provide a decision-making basis for land resource management in strategic adjustment among agricultural, social economic, ecological, energy, and sustainable land use. The remaining sections of the paper are organized as follows. In Section 2, the background of the research area and the data source and processing are introduced. Section 3 describes the methods and procedures, including theoretical framework, calculation method, and analysis process. Finally, Section 4 presents conclusions and some related policy implications.

## 2. Materials and Methods

### 2.1. Study Region

**Figure 1:**
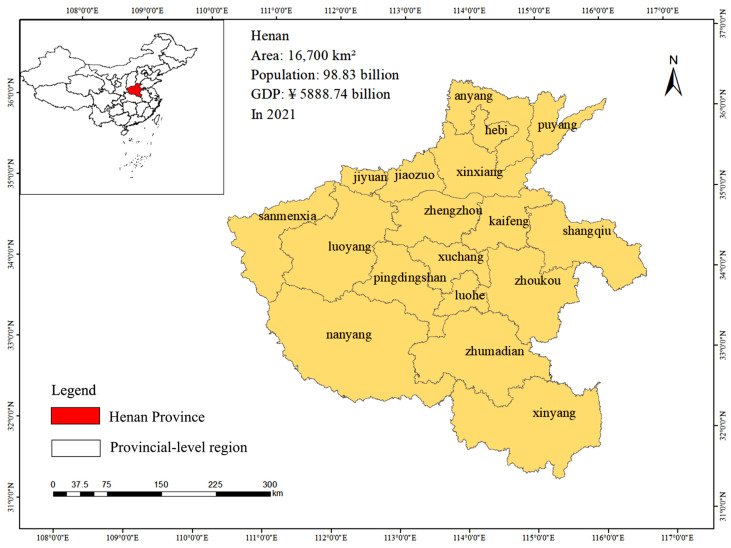
Location of the study region.

Henan Province (110°21′ E–116°39′ E, 31°23′ N–36°22′ N) is located in central-eastern China, in the middle and lower reaches of the Yellow River (Figure 1). It consists of 18 cities with a total area of 167,000 square kilometers, and in 2021 had a total population of 98.83 million (ranked third in China) and GDP of CNY 5,888.74 billion (ranked fifth) (HSB, 2021 [30]). Henan Province, with a cultivated area of 7.514 million hectares, mainly in the form of plains and basins, is the only province in China that crosses the Yangtze, Huaihe, Yellow, and Haihe River basins. The eight types of land use in Henan Province are cultivated land (49.49% of the total land area), forest land (21.19%), residential land (12.4%), garden land (1.4%), grassland (4.11%), transportation land (2.6%), water area (6.36%), and other land (2.46%).

### 2.2. Data Sourcesand Processing

Based on data accessibility and integrity, the research period of this study is set as 2010–2020. Basic data mainly include the following categories: (1) Land use data of Henan Province is from the Natural Resources Bulletin of Henan Province (DNRHP. [31]). (2) The selected data of ecological environment, social economy, agricultural production, and energy consumption of Henan Province come from the Bulletin of National Economic and Social Development of Henan Province (HSB. [32]), the Statistical Yearbook of Henan Province (HSB. [30]), and the Natural Resources Bulletin of Henan Province (DNRHP. [31]). (3) The remote sensing data of changes in land use types of 2010, 2015, and 2020 with spatial resolution of 1 km × 1 km in Henan Province were collected from the Cloud Platform of Resource and Environment Science and Data Center, Institute of Geographic Sciences and Natural Resources Research, Chinese Academy of Sciences.

We first use the 1 km×1 km spatial grid data of remote sensing images of Henan Province in 2010, 2015, and 2020, which we interpret as the basic unit to perform evaluation of the land use type transformation (LUTT). According to the existing statistical data (NBSC. [33]), the land use types can be divided into eight categories, namely, cultivated land, garden land, forest land, grassland, wetland, land used for urban, rural, industrial, and mining activities, land used for transport, land used for water, and water conservancy facilities. Then, the spatial-temporal evolution characteristics and influencing factors of coupling coordination among multiple land use efficiencies in agriculture, socio-economy, and ecology are further analyzed [29]. Furthermore, the land use performance is comprehensively evaluated according to the data of the social economy, ecological environment, agricultural production, and energy consumption in the Statistical Yearbook.

## 3. Methods and Procedures

### 3.1. Theoretical Framework

**Figure 2:**
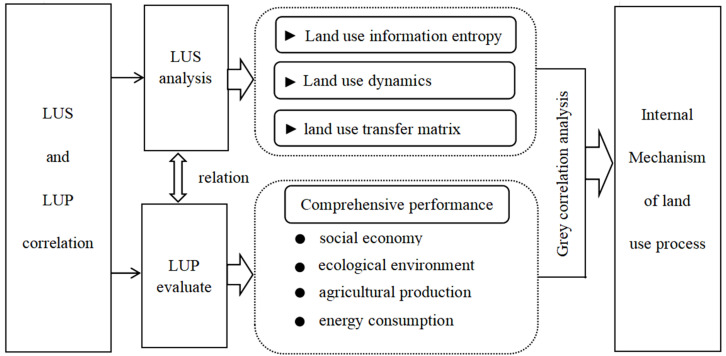
Analytical framework.

Land use and land cover change (LUCC) is the result of interaction between natural and human factors [7]. From the perspective of land use structure (LUS) change and land use performance (LUP), this paper studies the characteristics of LUS and its correlation with LUP in Henan Province, and then studies the coordination between land use and social economic development and environment. The analytical framework for the research is shown in Figure 2. First, the area data of different land use types in Henan Province from 2010 to 2020 were selected to calculate the information entropy, to then analyze the dynamic degree of land use. At the same time, ArcGIS was used to extract the land remote sensing images of Henan Province during the research period, and the land use transfer matrix was calculated to obtain the transformation of land types and the change process of LUS. Second, we integrated the social economy, ecological environment, agricultural production, and energy consumption to build a comprehensive evaluation model of LUP in Henan Province. LUP evaluation is the basic goal of promoting the optimal allocation of land resources and realizing the rational utilization of land resources, which is conducive to the policy formulation of strengthening land use and optimizing land supply structure. Then, the correlation between LUS and LUP in Henan Province was analyzed using the grey relational degree model. Finally, through the above research work, the internal mechanism between land use transformation and social economic development in Henan Province was revealed. This enables rational planning of the LUS in Henan Province, takes full advantage of the change in land use types in Henan Province to drive the transformation of the industrial structure, and achieves better development of the social economy, ecological environment, and agricultural production.

### 3.2. Dynamic Characteristics of Land Use Types

#### 3.2.1. Information of Land Use

A land use system is complicated and associated with nature, society, economy, production, and consumption [34,35]. It is also a typically controllable and open dissipative system with specific structures and functions [35,36]. Information entropy of land use structure (IE_LUS_) is a commonly used indicator [35] to measure the order degree of a regional land use system and determine its evolutionary direction [35]. The more orderly a system, the lower the information entropy; conversely, the more chaotic the system, the higher the information entropy. The order and equilibrium degree of the land use system can be described by information entropy:

(1) $${\mathrm{IE}}_{\mathrm{LUS}}=-\overset{n}{\underset{i=1}{\sum }}p_{i}\ln p_{i}$$

where IE_LUS_ is the information entropy of land use structure; the larger the information entropy, the more complex the land type and its internal structure. $p_{i}=A_{i}/A$ denotes the proportion of land type *i* in total area *A*, $\overset{n}{\underset{i=1}{\sum }}p_{i}=1$; $A_{i}$ is area of the land type *i*; A is total area of a region, $A=\overset{n}{\underset{i=1}{\sum }}A_{i}$.

#### 3.2.2. Dynamic Change in Land Use

The dynamic degree of land use (DD_LU_) can intuitively reflect the range and rate of change in each land type during the specific study period, which helps in the study of the mechanism of the change in land use type. The formula is as follows:

(2) $${\mathrm{DD}}_{\mathrm{LU}}=\left(\left(U_{a}-U_{b}\right)/U_{a}\right)\times T^{-1}\times 100\%$$

where U_a_ and U_b_ represent the area of a land use type at the beginning and end of the study, respectively. *T* is the study period; the value of DD_LU_ is the annual change rate of some land use type, where the unit is year.

#### 3.2.3. Conversion of Land Use Types

The land use transfer matrix is the main tool for specifically and quantitatively reflecting the structural characteristics of land use change and the mutual conversion direction of various land use types. Through the interact module of ArcGIS and the data perspective of Excel, the interpreted remote sensing images of 2010, 2015, and 2020 were folded and analyzed to obtain the land use transfer matrices of 2010–2015 and 2015–2020.The land use transfer matrix is as follows:

$$T_{ij}={\left[\begin{array}{cccc} T_{11} & T_{12} & \cdots & T_{1n}\\ T_{21} & T_{22} & \cdots & T_{2n}\\ \vdots & \vdots & \ddots & \vdots \\ T_{n1} & T_{n2} & \cdots & T_{nn} \end{array}\right]}_{n\times n}$$

where *T_ij_* is the land area converted from category *i* to *j* during the study period. *n* represents the land use types. The sum of each row of the transfer matrix represents the total area of type *i* land use at the beginning of the study, and the value of each row represents the transfer purpose and scale of land type.

### 3.3. Evaluation of Land Use Performance

#### 3.3.1. Construction of Comprehensive Evaluation Index System

Land use performance is a systematic representation of land resource utilization mode and benefit. It is led by the government with the aim of building scientific and sustainable land use patterns and achieving maximum social and economic benefits under the premise of maintaining a good ecological environment. In terms of the selection of evaluation indexes, the evaluation of land use performance has gradually developed from a single indicator reflecting the efficiency of a certain aspect to a multi-index comprehensive system including society, the economy, and the environment [1]. Combined with the actual land use situation in Henan Province, this study selected 20 indicators from four criteria layers of social economy (SE), ecological environment (EE), agricultural production (AP), and energy consumption (EC) to construct a comprehensive evaluation index system (Table 1) for regional land use performance measurement, and then explored the effects of land use and existing problems in Henan Province.

#### 3.3.2. Evaluation Model of Land Use Performance

(1) Standardized processing of the index original data

In order to eliminate the dimensional difference between different indicators that make comprehensive evaluation impossible, the standardized formulas of the original data are as follows:

The positive indicator data are represented by:

(3) $$x_{ij}^{\prime }=\frac{x_{ij}-x_{jmin}}{x_{jmax}-x_{jmin}}$$

The negative indicator data are represented by:

(4) $$x_{ij}^{\prime }=\frac{x_{jmax}-x_{ij}}{x_{jmax}-x_{jmin}}$$

where $x_{ij}^{\prime }$ is the standardized index value of the *j*-th evaluation index in the *i*-th year; $x_{ij}$ is its original value; $x_{jmax}$ and $x_{jmin}$ are the maximum and minimum initial values, respectively, of the *j*-th index in all years.

(2) Determination of index weight

To avoid the homogenization defect of the single weight method, the combinatorial method of the entropy weight and variation coefficient is used to assign weights.

The entropy weight formula is as follows:

(5) $$D_{j}=\frac{1-M_{j}}{m-\sum_{j=1}^{m}M_{j}}\;\mathrm{and}\;M_{j}=-\frac{1}{lnn}\sum_{i=1}^{n}b_{ij}lnb_{ij}$$

where $D_{j}$ is the entropy weight value of the *j*-th index; $0<D_{j}<1$ and $\overset{m}{\underset{j=1}{\sum }}D_{j}=1$. $M_{j}$ is the entropy value of the *j*-th index calculated by the information entropy.$b_{ij}=Y_{ij}/\overset{n}{\underset{i=1}{\sum }}Y_{ij}$ is the proportion of the *j*-th indicator in the *i*-th year ($b_{ij}lnb_{ij}=0$ if $b_{ij}=0$); $Y_{ij}$ is its standard value; *i*=1, 2, …, *n*, *j*=1, 2, …, *m*.

The weight formula of the variation coefficient is as follows:

(6) $$W_{j}=V_{j}/\sum_{j=1}^{m}V_{j}\;\mathrm{and}\;V_{j}=S_{j}/\bar{x_{j}}$$

where $W_{j}$ is the weight value of the *j*-th index determined by the variation coefficient. $V_{j}$ and $S_{j}$ are respectively the variation coefficient and standard deviation of the *j*-th index; $\bar{x_{j}}$ is the average of the *j*-th indexes, *j*=1, 2, …, *m*.

The combined weight is:

(7) $$N_{j}=\frac{D_{j}W_{j}}{\sum_{j=1}^{m}D_{j}W_{j}}$$

where $N_{j}$ is the combination weight value of the entropy weight and variation coefficient for the *j*-th index.

(3) Performance evaluation model

According to the principle of the multi-factor weighted score method, the evaluation model of regional land use performance is obtained as:

(8) $$C_{j}=\overset{m}{\underset{j=1}{\sum }}x_{ij}^{\prime }N_{j}$$

(9) $$P=\overset{m}{\underset{j=1}{\sum }}C_{j}N_{j}$$

where $C_{j}$ represents the land use performance scores, ranging from 0 to 1. $x_{ij}^{\prime }$ is the normalized value of the *j*-th indicator in the *i*-th year (i=1, 2, …, *n*;j=1, 2, …, m). *P* is the comprehensive performance value of land use. $N_{j}$ is the combined weight of each index.

### 3.4. Evaluation of the Correlation between LandUse Structure and Land Performance

Grey system theory is an interdisciplinary research field [37]. For land use systems, grey relational analysis (GRA) is mostly manifested in the interaction or relation between the elements of land resources [1,37]. Its basic idea is the geometric similarity of time series [1]. This research used GRA to analyze the grey correlation grade between the land use performance value, land use type, and information entropy, and to further describe the evolution pattern of the system [37]. The land use performance index $\left\{C\right\}=\left[C_{1},C_{2},\ldots ,C_{n}\right]$ is selected as the parent sequence, and the land use structure and its characterization value information entropy $\left\{C_{i}\right\}=\left[C_{i1},C_{i2},\ldots ,C_{in}\right]$, which affected the level of land use performance, are taken as the subsequence. Then, the correlation coefficient of the parent and subsequence at point *K* is as follows:

(10) $$\xi_{i}\left(k\right)=\frac{\underset{i}{\min}\underset{k}{\min}\left|C_{k}-C_{ik}\right|+\rho \underset{i}{\max}\underset{k}{\max}\left|C_{k}-C_{ik}\right|}{\left|C_{k}-C_{ik}\right|+\rho \underset{i}{\max}\underset{k}{\max}\left|C_{k}-C_{ik}\right|}$$

where $\xi_{i}$ is the correlation coefficient of *C* and *C_i_* at time *k*. $\left|C_{k}-C_{ik}\right|$ is the absolute difference between parent sequence and subsequence at time *k* (*k*= 1, 2…*n*). $\underset{i}{\min}\underset{k}{\min}\left|C_{k}-C_{ik}\right|$ and $\underset{i}{\max}\underset{k}{\max}\left|C_{k}-C_{ik}\right|$, respectively, are the minimum and maximum absolute difference at each time. ρ is the resolution coefficient, 0<ρ<1, The smaller the value, the higher the resolution;ρ=0.5 in this paper.

(11) $$r_{i}=\frac{1}{n}\overset{n}{\underset{k=1}{\sum }}\xi_{i}\left(k\right)$$

where $r_{i}$ is the grey relational degree; the closer the value to 1, the better the correlation.

## 4. Results and Discussion

### 4.1. Analysis of Information Entropy in Land Use Structure

The calculated information entropy (IE) of land use structure in Henan Province (Table 2) showed that the total trend increased gradually during the study period (2010–2020), but the rise was slower. (1) From 2010 to 2015, it increased slightly with an average annual increase of 0.16%. During this period, the area of cultivated land, forest land, garden land, and grassland decreased, while land used for urban, rural, industrial, and mining activities, transport, and water and water conservancy facilities increased significantly. The size of the city gradually increased. (2) The IE decreased slightly in 2016; however, it increased slightly again in 2017 and increased by 8.465% in 2018. That is, in the past two years, the order degree of land use structure in Henan Province decreased. Facts also indicate that the area of cultivated land was reduced by 19,600 hectares because of construction occupation of land such as transportation, water and water conservancy facilities, disaster-destroyed farmland, and agricultural structure adjustment. (3) From 2018 to 2020, the IE of land use structure gradually declined slightly, with an average annual decrease of 0.74%. This is largely because the pattern of the cultivated land protection as a priority in Henan Province was established against the background of the land management regulations proposed by the Ministry of Land and Resources to improve land use efficiency and increase the balance of land use structure. The pattern comprehensively applied administrative, economic, technical, and legal factors, among others, to manage and control the cultivated land and implement the balance system of cultivated land occupation and compensation.

To sum up, during the study period, the range of the change in land use information entropy (IE) in Henan Province is smaller and its structure is relatively stable. With the scientific planning of land resources by the government, the land type tends to be rationalized, and the IE value of land use will become smaller and smaller.

### 4.2. Analysis of Land Use Change Rate

**Figure 3:**
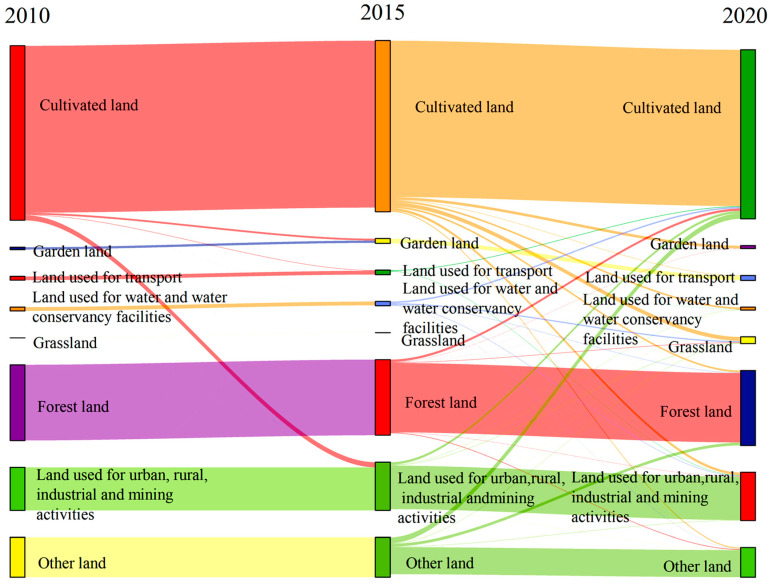
Area change in various types of land use in Henan Province from 2010 to 2020.

Figure 3 shows the area change in various land types in Henan Province from 2010 to 2020. Among all the land types in Henan Province, cultivated land occupies a large share (accounting for more than 40% of the total area), but from 2010 to 2020, the cultivated land area shrank year by year and gradually transformed into other types of land, such as increased land used for water and water conservancy facilities, transportation, and grassland. The range of forest land change is not significant. The area of other land was reduced by 11.75%, and mainly converted into part of the cultivated land and forest land. Thus, the cultivated land and forest land was still the main land type in Henan Province at the end of 2020.

**Figure 4:**
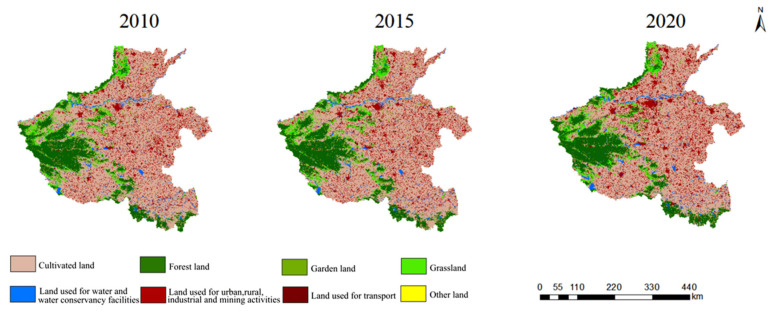
Land type change in Henan Province in 2010, 2015, and 2020.

According to Formula (3) the average annual change rates of each land use type over five and ten years from 2010 to 2020 can be calculated (Table 3). The results showed that: (1) The fastest changing rate in ten years was land used for water and water conservancy facilities, followed by land used for transport, garden land, other land, and forest land. Simultaneously, the annual change rate of land used for urban, rural, industrial, and mining activities was relatively small, and there was a trend of a year-by-year decrease in cultivated land. (2) From 2010 to 2015, the largest change was in land used for transport, with an annual growth rate of 4.22%. It was followed by land used for urban, rural, industrial, and mining activities, and water and water conservancy facilities, with annual growth rates of 1.34% and 0.35% respectively. The smallest changes were in cultivated land and forest land, with an annual rate of decrease of 0.18%. (3) From 2015 to 2020, the annual increase in land used for water conservancy facilities reached a rate of 71.05%, which is principally due to the policy implemented in Henan Province in 2017 of returning farmland to forest and grassland, and obviously speeding up the construction and reinforcement of water conservancy facilities, and improving soil and water conservation and water ecological protection. As a result, the vegetation coverage rate of Henan Province increased from 57.3% in 2000 to 66.2% in 2018 (Figure 4). The next obvious increase was in transport, garden land, and other land.

### 4.3. Analysis of Land Use Transfer Matrix

**Figure 5:**
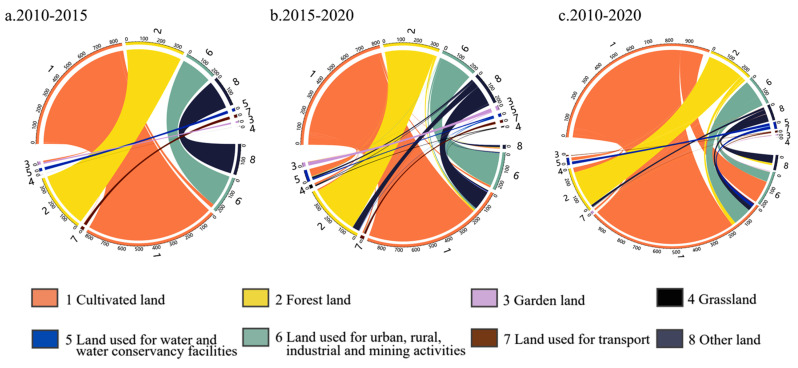
Spatial transformation of different land use types in Henan Province from 2010 to 2020 (10^2^ km^2^).

The land type transfer matrix cannot only reveal the changes in the area in each type of land use, but also enables a visual and clear judgment of the change trajectory of land types (Figure 5). (1) The land type change in Henan Province from 2010 to 2015 was primarily manifested by the increase in land used for transport, urban, rural, industrial, and mining activities. Most of these increases came chiefly from the cultivated land, with a transfer area of 756.43 km^2^. Other undeveloped land was transformed into land used for water and water conservancy facilities, and a small part of land was used for transport. (2) The key change in land types from 2015 to 2020 was the transfer of cultivated land to land used for urban, rural, industrial, and mining activities (91.87 km^2^), transport land (726.38 km^2^), and garden land (188.07 km^2^). Other land mainly transformed into land used for water and water conservancy facilities. Overall, in the past decade, all converted land mainly came from the reduction in cultivated land and other land. (3) From 2010 to 2020, the general trend is that cultivated land was mainly converted into land used for urban, rural, industry, and mining activities, water area and water conservancy facilities, and grassland. Other land was mainly converted into forest land. The land for water area and water conservancy facilities increased substantially, mainly from cultivated land.

### 4.4. Analysis of Land Use Performance

**Figure 6:**
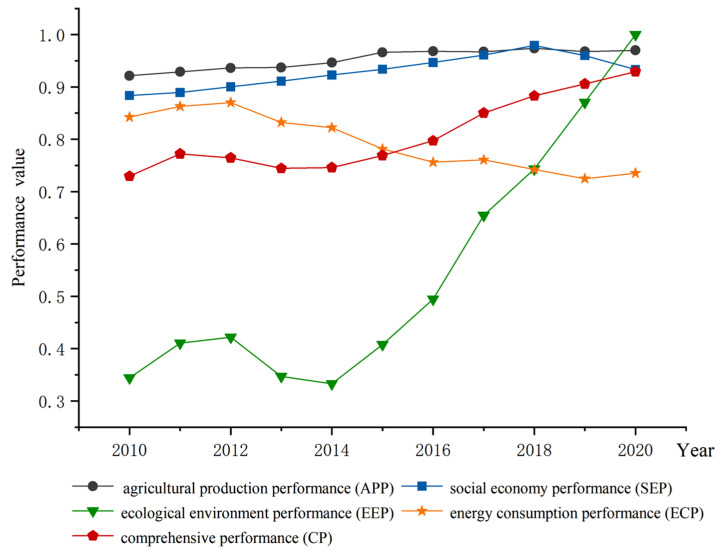
Changes in land use performance in Henan Province from 2010 to 2020.

Through the calculation of the regional land use performance evaluation model, the performance values of agricultural production, social economy, ecological environment, and energy consumption, and the comprehensive performance in Henan Province from 2010 to 2020 were obtained (Figure 6).

#### 4.4.1. Agricultural Production Performance (APP)

APP is in a steady and slow rising state. As a major grain-producing province, Henan Province has high agricultural production efficiency, so it reached 0.921 in all types of land use performance in 2010, and reached a maximum of 0.97 in 2020. The agricultural performance fluctuated slightly from 2017 to 2020. Nevertheless, the overall trend was still increasing, mainly because the sown area and land reclamation rate decreased slightly year by year in this period. This also reflects the fact that, since 2017, part of the cultivated land in Henan Province has been transformed into construction land, but at the same time, agricultural production technology and efficiency have been greatly improved to a certain extent.

#### 4.4.2. Eco-Environmental Performance (EEP)

EEP was the lowest among the four performance indicators at the beginning of the study period, with a performance value of 0.344 in 2010. There was even a decline between 2013 and 2014. This was mainly due to a decrease in ecological and environmental water replenishment (from 669 million m^3^ to 566 million m^3^). However, it was the fastest growing performance from 2014 onward, and reached the maximum among all performances at the end of the study period. In 2017, the work of returning farmland to forest played an increasingly significant role in conserving water sources, wind prevention and sand fixation, and improving the ecological environment, and achieved good results. In recent years, Henan Province has also augmented the construction of urban environmental infrastructure, enhanced the disposal capacity of domestic waste, and reduced environmental pollution. While controlling pollution in key industries, Henan province has also enlarged the per capita public green space and coverage in urban areas, which reached 14.4m^2^ and 25.1% by 2020, respectively. These combinational measures rapidly promoted the benefits of the ecosystem. As a result, the environmental quality of Henan Province has been continuously improved.

#### 4.4.3. Socio-Economic Performance (SEP)

The development trend of SEP is basically similar to that of APP, which is mainly related to the fact that Henan Province is a major agricultural province, and agriculture plays a pivotal role in the economic development structure. SEP reached the highest value in 2018, and its proportion of tertiary industry increased from 28.1% in 2010 to 42.7%. Accompanied by optimization and adjustment of the industrial structure, SEP of Henan Province has been greatly improved in the past decade. With the growth in population, and effective government support of employment, medical insurance, housing, etc., the output values of industry and agriculture have been improved year by year. Performance values dropped to 0.959 and 0.933 between 2019 and 2020, respectively, because of the COVID-19 epidemic, but Henan Province’s economy has still maintained a steady and sustained positive trend.

#### 4.4.4. Energy Consumption Performance (ECP)

Overall, ECP showed a downward trend. Since energy consumption per unit GDP decreased by 13.708% from 2010 to 2012, the performance value (inverse index) increased from 0.842 to the maximum of 0.87. ECP presented a fluctuating decline from 2013 to 2020, with an average annual decrease of 0.017. Although energy consumption increased from 21.09 million tons of standard coal to 227.52 million tons of standard coal during this period, carbon emission intensity and energy consumption per unit GDP decreased year by year and reached the lowest value at the end of 2020, with an average annual decline of 3.025% and 5.054%, respectively. This reflects the increasing demand for energy in Henan Province because of population growth and economic development. Facing the arduous task of reaching a “carbon peak” before 2030 and the corresponding policies to reduce carbon emissions, Henan Province has achieved remarkable results in industrial and energy consumption structural adjustment and environmental governance. The increase in the new area of farmland returning to forests has enhanced the capacity of forest carbon sinks and alleviated the carbon emission problem. Consequently, the relationship between energy consumption and the ecological environment is inseparable. That is, effective ecological environment treatment can relieve the pressure of carbon emissions.

#### 4.4.5. Comprehensive Performance (CP)

The CP of land use in Henan Province increased from 0.73 in 2010 to 0.929 in 2020, and the performance level has been continuously improved, with the maximum increase from 2016 to 2017 being 0.053. This is attributed to the implementation of the concept of green coordinated development in Henan Province in recent years. The various policies issued pay more attention to the sustainable and harmonious development of humans and nature, which continuously strengthens the coordination of the elements within the land use system.

### 4.5. Correlation Analysis between Land Use Structure and Performance

The grey correlation method (Equations (10) and (11)) was used to calculate the change in land use structure and its information entropy in Henan Province from 2010 to 2020. The grey correlation classification is: 0–0.6 is a relatively weak correlation; 0.6–0.8 indicates general correlation; and 0.8–1 is a relatively strong correlation [1]. Generally, a grey correlation degree of greater than 0.6 indicates that there is an obvious correlation between land use structure (LUS) and land use performance (LUP). That is, the closer the relationship, the greater the impact on land structure.

**Figure 7:**
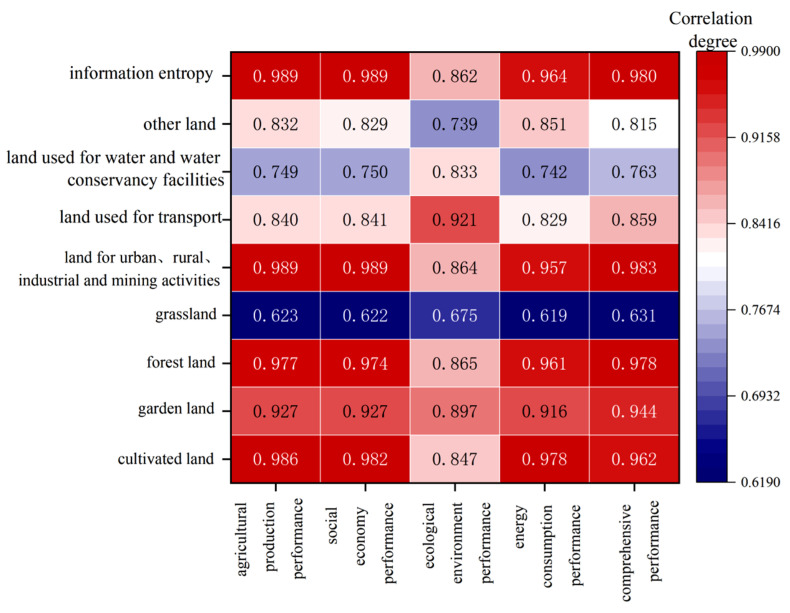
Correlation degree between land use structure and performance in Henan Province.

From the calculation results (Figure 7), the correlation degree between land use structure (LUS) and performance of social economy (SE), ecological environment (EE), agricultural production (AP), and energy consumption (EC) was relatively close. During the research period, the IE of land use structure was increasing year by year, indicating that the stability of land use types and rationality of land use structure in Henan Province were reinforced.

#### 4.5.1. Correlation between APP and LUS

The correlations between agricultural benefits and land use structure (LUS) in Henan Province were, in order, information entropy (0.989), land used for urban, rural, industrial, and mining activities (0.989), cultivated land (0.986), forest land (0.977), garden land (0.927), land used for transport (0.84), and other land (0.832). As a major agricultural province, these correlations between agricultural production performance (APP) and LUS show that with the expansion in urban construction land in recent years, the occupation of agricultural land has become more prominent, which should be of great concern.

#### 4.5.2. Correlation between SEP and LUS

The correlation degree between socio-economic performance (SEP) and LUS was consistent with that mentioned above of APP and LUS. Urban expansion was closely related to social and economic development, and the cultivated land and the forest land also had a strong correlation with the social economy. All these results indicate that social development has a relatively significant impact on land structure.

#### 4.5.3. Correlation between EEP and LUS

Ecological environment is a sign of the benign development of land utilization. During the study period, from the ecological performance associated with LUS, there was a strong correlation degree in land used for transport (0.921), garden land (0.897), forest land (0.865), land used for urban, rural, industrial, and mining activities (0.864), information entropy (0.862), cultivated land (0.847), land used for water and water conservancy facilities (0.833), and other land (0.739), and grass land had a general correlation (0.675). On the basis of the relational degree between ecological performance and LUS, the transportation and green space coverage have a significant influence on the ecological environment.

#### 4.5.4. Correlation between ECP and LUS

The strong correlation degree between energy consumption and LUS, ranked from large to small, was: cultivated land (0.978), information entropy (0.964), forest land (0.961), land used for urban, rural, industrial, and mining activities (0.957), garden land (0.916), other land (0.851), and land used for transport (0.829); land used for water and water conservancy facilities (0.742) and grassland (0.62) were generally correlated. It can be seen from this correlation that cultivated land, green space, urban construction, and industrial and mining production activities are closely related to energy consumption. That is, as a major agricultural province, Henan Province has a large demand for energy from agricultural mechanized production, urban construction, and industrial and mining activities.

#### 4.5.5. Correlation between CP and LUS

The calculation results of grey relational analysis (GRA) show that the relationship between comprehensive performance and LUS is as follows: land used for urban, rural, industrial, and mining activities (0.983), information entropy (0.98), forest land (0.978), cultivated land (0.962), garden land (0.944), land used for transport (0.859), other land (0.816), land used for water and water conservancy facilities (0.763), and grassland (0.631). Compared with the other subsystems mentioned above, it was found that the correlation degree between urban construction, industrial, mining, and agricultural activities, forestry and garden land, and LUP is greater than 0.9, indicating that industrial and agricultural production has a strong response to land use. The correlation between grassland, water area, and LUP was always at a general level; that is, they make little contribution to the benefits.

**Figure 8:**
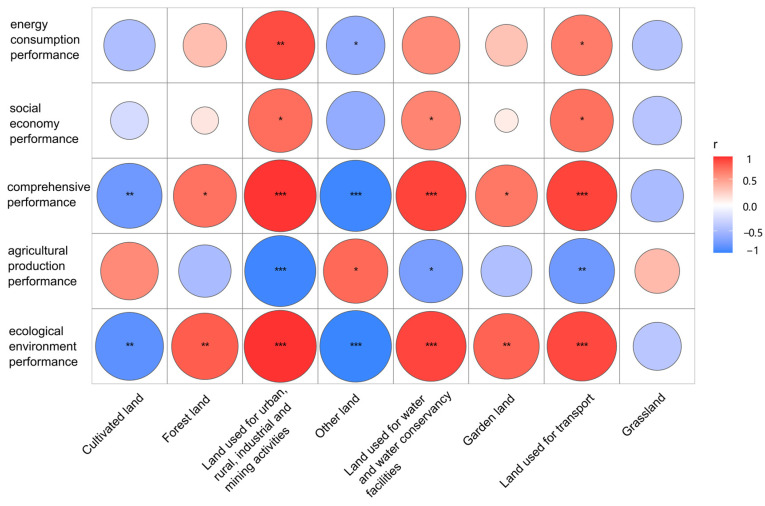
Correlation analysis results of LUS and LUP (***: *p* ≤ 0.001; **: 0.001< *p* ≤ 0.01; *: 0.01< *p*< 0.05; unmarked: *p* ≥ 0.05).

From the perspective of the correlation between various subsystems and LUP, Henan Province has a significant impact on land resources in urbanization construction and land used for industrial and mining enterprises. At the same time, the ecological environment has also been greatly improved through the policy of returning farmland to forests. Through correlation analysis and the significance test, the results (Figure 8) show that there was a significant correlation between land use type and land performance. The ecological environment performance (EEP) and comprehensive performance (CP) were positively correlated with forest land, garden land, land used for urban areas and villages, industrial and mining activities, water area and water conservancy facilities, and transportation, but significantly negatively correlated with cultivated land and other land. There was no significant correlation between socio-economic performance (SEP) and energy consumption performance (ECP) on forest land and garden land.

## 5. Conclusions

In this study, analysis of land use type transformation, information entropy, and grey relational analysis were applied to estimate the efficiency and order of land use structure and their grey relational grade from the perspective of the social economy, ecological environment, agricultural production, and energy consumption performance in Henan Province of China from 2010 to 2020. Given this assessment, the conclusions are as follows.

Based on the matrix analysis of land use type transformation, in the land use structure of Henan Province in the past ten years, among all land types, cultivated land occupies the largest proportion, although it decreased year by year and transformed into other land uses. In terms of the land change rate, land used for water and water conservancy facilities, transport, urban, rural, industrial, and mining activities, and other land, are more significant. Although different land use types changed to different degrees during the study period, the information entropy of the land use system in Henan Province fluctuated little and its stability gradually strengthened. In land use performance (LUP), in the past decade, Henan Province achieved the best grade in the ecological environment, but the lowest score in terms of energy consumption. In addition, the comprehensive performance showed an upward trend. According to the correlation degree between land use structure (LUS) and land use performance (LUP), there was a significant correlation between the performance of each subsystem and land use types (>0.6) during the study period. Moreover, agricultural production and social economy are closely related to cultivated land, forest land, and land used for urban, rural, industrial, and mining activities, while the correlation degree with other land is the lowest. This indicates that the structure of land type has significant influence on the performance of land use.

## 6. Policy Implications

As a major agricultural, economic, and populous province in Central China, Henan Province has the most active economic activities and outstanding contradictions between human and land resources. It is necessary to study the change characteristics of land use types and land use performance, which plays an imperative role in guiding the sustainable use of land resources and ensuring the benign interaction between the environment and social economy. As two essential dimensions of land use evaluation, structure and efficiency of land use have a momentous impact on regional sustainable development. The research identified that several problems were found in the land use of Henan Province during its development, for example, the conversion of cultivated land to construction land, low efficiency and high emission of energy use, and the unbalanced development of the east and the west. As a major grain-producing province in China, Henan Province plays a crucial role in national food security, so it is necessary to scientifically plan agricultural production and continuously optimize the structure of industrial and agricultural land use. Furthermore, in the process of boosting the market-oriented allocation of land resources in the future, it is necessary to advance the efficiency of land use while obtaining land income.

Judging from the ecological performance of land use, Henan Province has made good achievements in ecological environment governance in the past decade, which is inseparable from the external effect of the implementation of ecological environmental protection policies on land type adjustment. With the implementation of various land policies in Henan Province, especially returning farmland to forests and developing of clear water areas and green mountains, the change in land types has been accelerated. The adjustment of these forms of land cover not only achieved obvious ecological benefits, but also enhanced the comprehensive performance of land use as a whole. At the same time, they have also played a role in promoting the social economy and sustainable development. These factors further confirm the vital importance of guiding policies for the optimal allocation and sustainable utilization of land resources. In the future, we should further optimize the energy and industrial structure, strengthen environmental monitoring, and execute measures such as cleaner production, energy conservation, and emission reduction.

## Figures and Tables

**Table 1:** Comprehensive evaluation index system of land use performance in Henan Province.

Target Layer	Criteria Layer	Index Layer	Weight
		Secondary and tertiary industries share of the contributions to the increase in GDP (%)	0.006
		Population density (person/sq.km)	0.004
	SE	Average land of investment in fixed assets in the whole province (10,000 CNY/km^2^)	0.108
		Average land of GDP (10,000 CNY/km^2^)	0.07
		Average land of Gross Output Value of Agriculture (10,000 CNY/km^2^)	0.811
		Coverage rate of green areas developed (%)	0.042
		Harmless transport and disposal of consumption waste (10,000 Tons)	0.039
	EE	Ecological environment replenishment (100 million m^3^)	0.721
		Per capita green area (sq.m)	0.174
		Forest-coverage rate (%)	0.023
Land use performance		Total sown area (1000 hectares)	0.055
		Grain output (10,000 Tons)	0.239
	AP	Land reclamation rate (Area of regional cultivated land/Total area of regional land, %)	0.371
		Multiple crop index (Total sown areas of farm crops/Cultivated land, %)	0.19
		Irrigated area of cultivated land (1000 hectares)	0.146
		Total energy consumption (10 thousand Tons of SCE (Standard Coal Equivalent))	0.046
		Carbon emission intensity (Carbon footprint/GDP, Tons/10,000 CNY)	0.246
	EC	Agriculture energy consumption (10 thousand Tons of SCE)	0.108
		Annual per capita consumption for households (kg of SCE)	0.238
		Per-unit GDP of total energy consumption (Tons/10,000 CNY)	0.361

**Table 2:** Calculated results of land use information entropy in Henan Province.

Year	2010	2011	2012	2013	2014	2015	2016	2017	2018	2019	2020
entropy value	1.36	1.36	1.37	1.37	1.37	1.37	1.37	1.37	1.49	1.48	1.47

**Table 3:** Average annual change rate of land use in Henan Province. (%).

Land Use Type	Cultivated Land	Garden Land	Forest Land	Grassland	Land Used for Urban, Rural, Industrial, and Mining Activities	Land Used for Transport	Land Used for Water and Water Conservancy Facilities	Other Land
2010–2015	−0.18	−0.80	−0.18	−1.77	1.34	4.22	0.35	−0.52
2015–2020	−1.46	18.79	5.33	−0.53	2.07	22.11	71.05	−14.79
2010–2020	−0.82	8.62	2.55	−1.13	1.78	15.50	36.32	−7.46

## Data Availability

No new data were created or analyzed in this study. Data sharing is not applicable to this article.

## References

[1] Fu Y.H., Zhou T.T., Yao Y.Y., Qiu A., Wei F.Q., Liu J.Q., Liu T. (2021). Evaluating efficiency and order of urban land use structure: An empirical study of cities in Jiangsu, China. J. Clean. Prod..

[2] Wu H., Lin A.Q., Xing X.D., Song D.X., Li Y. (2021). Identifying core driving factors of urban land use change from global land cover products and POI data using the random forest method. Int. J. Appl. Earth Obs..

[3] Ding T., Yang J., Wu H.Q., Liang L. (2022). Land use efficiency and technology gaps of urban agglomerations in China: An extended non-radial meta-frontier approach. Socio-Econ. Plan. Sci..

[4] Wang L.Y., Zhang S.Y., Tang L.P., Lu Y.C., Liu Y.F., Liu Y.L. (2022). Optimizing distribution of urban land on the basis of urban land use intensity at prefectural city scale in mainland China. Land Use Policy.

[5] Xu F., Wang Z.Q., Chi G.Q., Wang D.H., Zhang Z.X., Zuo D.Y. (2021). Differentiation and progress of urban regionalization in China: Perspectives of land use and geography. Appl. Geogr..

[6] Zuo Q., Zhou Y., Wang L., Li Q., Liu J.Y. (2022). Impacts of future land use changes on land use conflicts based on multiple scenarios in the central mountain region, China. Ecol. Indic..

[7] Zhou Y., Li X.H., Liu Y.S. (2020). Land use change and driving factors in rural China during the period 1995–2015. Land Use Policy.

[8] Shi X.Y., Matsui T., Machimura T., Haga C., Hu A., Gan X.Y. (2022). Impact of urbanization on the food–water–land–ecosystem nexus: A study of Shenzhen, China. Sci. Total Environ..

[9] Shen Z.Y., Wang Y.F., Su H., He Y., Li S. (2022). A bi-directional strategy to detect land use function change using time-series Landsat imagery on Google Earth Engine: A case study of Huangshui River Basin in China. Sci. Remote Sens..

[10] Xu H.T., Song Y.C., Tian Y. (2022). Simulation of land-use pattern evolution in hilly mountainous areas of North China: A case study in Jincheng. Land Use Policy.

[11] Huang X.X., Wang H.J., Xiao F.T. (2022). Simulating urban growth affected by national and regional land use policies: Case study from Wuhan, China. Land Use Policy.

[12] Burra D.D., Parker L., Than N.T., Phengsavanh P., Long C.T.M., Ritzema R.S., Sagemueller F., Douxchamps S. (2021). Drivers of land use complexity along an agricultural transition gradient in Southeast Asia. Ecol. Indic..

[13] Zhang X.B., Timo de Vries W., Li G., Ye Y.M., Zhang L.L., Huang H.L., Wu J.Y. (2021). The suitability and sustainability of governance structures in land consolidation under institutional change: A comparative case study. J. Rural Stud..

[14] Luo X., Lu X.H., Jin G., Wan Q., Zhou M. (2019). Optimization of urban land-use structure in China’s rapidly developing regions with eco-environmental constraints. Phys. Chem. Earth.

[15] Tian Y.Y., Zhou D.Y., Jiang G.H. (2021). A new quality management system of admittance indicators to improve industrial land use efficiency in the Beijing-Tianjin-Hebei region. Land Use Policy.

[16] Liu H.J., Yan F.Y., Tian H. (2022). Towards low-carbon cities: Patch-based multi-objective optimization of land use allocation using an improved non-dominated sorting genetic algorithm-II. Ecol. Indic..

[17] Lei W.Q., Jiao L.M., Xu G. (2022). Understanding the urban scaling of urban land with an internal structure view to characterize China’s urbanization. Land Use Policy.

[18] Schiavina M., Melchiorri M., Freire S., Florio P., Ehrlich D., Tommasi P., Pesaresi M., Kemper T. (2022). Land use efficiency of functional urban areas: Global pattern and evolution of development trajectories. Habitat Int..

[19] He X.P. (2022). Energy effect of urban diversity: An empirical study from a land-use perspective. Energy Econ..

[20] Chen W., Chen W.J., Ning S.Y., Liu E.N., Zhou X., Wang Y.N., Zhao M.J. (2019). Exploring the industrial land use efficiency of China’s resource-based cities. Cities.

[21] Dong Y., Jin G., Deng X.Z. (2020). Dynamic interactive effects of urban land-use efficiency, industrial transformation, and carbon emissions. J. Clean. Prod..

[22] Chen W., Su Z., Wang Y.N., Wang Q., Zhao G.L. (2022). Do the rank difference of industrial development zones affect land use efficiency? A regional analysis in China. Socio-Econ. Plan. Sci..

[23] Li H.Q., Zhao Y.Y., Zheng F. (2020). The framework of an agricultural land-use decision support system based on ecological environmental constraints. Sci. Total Environ..

[24] Ustaoglu E., Sisman S., Aydınoglu A.C. (2021). Determining agricultural suitable land in peri-urban geography using GIS and Multi Criteria Decision Analysis (MCDA) techniques. Ecol. Model..

[25] Gao X., Zhang A., Sun Z. (2020). How regional economic integration influence on urban land use efficiency? A case study of Wuhan metropolitan area, China. Land Use Policy.

[26] Song Y., Yeung G., Zhu D.L., Xu Y., Zhang L.X. (2022). Efficiency of urban land use in China’s resource-based cities, 2000–2018. Land Use Policy.

[27] He S.W., Yu S., Li G.D., Zhang J.F. (2020). Exploring the influence of urban form on land use efficiency from a spatiotemporal heterogeneity perspective: Evidence from 336 Chinese cities. Land Use Policy.

[28] Zhang L., Zhang L., Xu Y., Zhou P., Yeh C. (2020). Evaluating urban land use efficiency with interacting criteria: An empirical study of cities in Jiangsu China. Land Use Policy.

[29] Liu J., Jin X.B., Li H.B., Zhang X.L., Xu W.Y., Fan Y.P., Zhou Y.K. (2022). Spatial-temporal changes and driving factors of the coordinated relationship among multiple land use efficiencies integrating stakeholders’ vision in eastern China. J. Clean. Prod..

[30] Henan Statistics Bureau (HSB) (2021). Henan Statistical Yearbook 2011–2021.

[31] Department of Natural Resources of Henan Province (DNRHP) (2021). Natural Resources Bulletin of Henan Province 2011–2021.

[32] Henan Statistics Bureau (HSB) (2021). Bulletin of National Economy and Social Development of Henan Province 2011–2021.

[33] National Bureau of Statistics of China (NBSC) (2021). China Statistical Yearbook 2011–2021.

[34] Chen W., Chi G., Li J. (2019). The spatial association of ecosystem services with land use and land cover change at the county level in China, 1995–2015. Sci. Total Environ..

[35] Chen Y. (2020). Equivalent relation between normalized spatial entropy and fractal dimension. Physica A.

[36] Chen W.X., Zeng J., Li N. (2020). Change in land-use structure due to urbanization in China. J. Clean. Prod..

[37] Wang H., Zhang C., Yao X.C., Yun W.J., Ma J.N., Gao L.L., Li P.S. (2022). Scenario simulation of the tradeoff between ecological land and farmland in black soil region of Northeast China. Land Use Policy.

